# The Association of Postcardiac Surgery Acute Kidney Injury with Intraoperative Systolic Blood Pressure Hypotension

**DOI:** 10.1155/2013/174091

**Published:** 2013-11-14

**Authors:** Solomon Aronson, Barbara Phillips-Bute, Mark Stafford-Smith, Manuel Fontes, Jeffrey Gaca, Joseph P. Mathew, Mark F. Newman

**Affiliations:** ^1^Department of Anesthesiology, Duke University Medical Center (DUMC) P.O. Box 3094, Baker House, Rm. 101, Durham, NC 27710, USA; ^2^Department of Surgery, Cardiovascular Division, Duke University Medical Center (DUMC) P.O. Box 3094, Baker House, Rm. 101, Durham, NC 27710, USA

## Abstract

*Background*. Postoperative acute kidney injury (AKI) is associated with high mortality and substantial cost after aortocoronary bypass graft (CABG) surgery. We tested the hypothesis that intraoperative systolic blood pressure variation is associated with postoperative AKI. *Methods*. We gathered demographic, procedural, blood pressure, and renal outcome data for 7,247 CABG surgeries at a single institution between 1996 and 2005. A development/validation cohort methodology was randomly divided (66% and 33%, resp.). Peak postoperative serum creatinine rise relative to baseline (%ΔCr) was the primary AKI outcome variable. Markers reflective of intraoperative systolic blood pressure variation were derived for each patient including (1) peak and nadir values (absolute and relative to baseline) and (2) excursion episodes beyond selected thresholds (by duration, frequency, and duration × degree). Each marker of systolic blood pressure variation was then separately evaluated for association with AKI using linear regression models with adjustment for several known risk factors (age, aprotinin use, congestive heart failure, previous myocardial infarction, baseline creatinine, bypass time, diabetes, weight, concomitant valve surgery, gender, and preoperative pulse pressure). *Results*. An association was identified between systolic blood pressure relative to baseline and postoperative AKI (*P* < 0.006). *Conclusions*. In CABG surgery patients, intraoperative systolic blood pressure decrease relative to baseline systolic blood pressure is independently associated with postoperative AKI.

## 1. Introduction

 It is commonly purported that poor perioperative hemodynamic control during cardiac surgery leads to postoperative acute kidney injury (AKI) [1–9]. Systolic blood pressure and pulse pressure amplification are due to changes in arterial stiffness that affect wave propagation along the arterial tree. Both systolic blood pressure and pulse pressure are higher in the periphery than in the central arteries for the same mean arterial pressure (MAP) and diastolic blood pressure. Whereas MAP refers exclusively to steady pressure, vascular resistance, and small arteries, systolic blood pressure and pulse pressure refer to pulsatile pressure and are determined by stroke volume, arterial stiffness, and wave reflection.

Whereas a large rise in serum creatinine (≥100%) over baseline has been shown to portend a doubling of in-hospital mortality, it has also been shown that even the small relative increases in creatinine, used as a sensitive index of AKI after aortocoronary bypass graft (CABG) surgery, can be associated with higher mortality and substantial additive cost [10–18]. 

 Cardiac surgery-associated AKI appears mechanistically to be related to preexisting renal dysfunction, diabetes and glucose intolerance, ventricular dysfunction, older age, preexisting hypertension, micro- and macroembolic processes, inflammatory mediators, prolonged duration of cardiopulmonary bypass (CPB), sensitivity to sympathetic stimulation, and perturbations of renovascular resistance and flow [3, 4, 6, 8–10, 19–23]. 

 Although preoperative characterizations of blood pressure such as systolic hypertension and widened pulse pressure are already known to be predictors of ischemic postoperative events including AKI [3, 4, 6, 8, 24–31], few data have characterized the relationship between intraoperative systolic blood pressure variability and postcardiac surgery AKI. Since even small postoperative serum creatinine elevations are strongly associated with increased mortality risk, [11–14, 32, 33] we tested the hypothesis that intraoperative systolic blood pressure variability is associated with postoperative AKI.

## 2. Methods

### 2.1. Study Population

With Institutional Review Board approval, we retrospectively studied a cohort of 7247 consecutive patients undergoing primary nonemergent CABG surgery procedures performed at Duke University Medical Center between September 1st, 1996, and December 30th, 2005. Demographic, perioperative, and renal outcome data, including patient characteristics, were accessed from the prospectively collected Duke Databank for Cardiovascular Diseases. The Databank is prospectively compiled from contemporaneous medical records, custom datasheets, and records of laboratory results. Database quality assurance involves random chart review for data confirmation and assessment of data completeness. In-hospital complications were classified using the Society of Thoracic Surgeons (http://www.sts.org/) criteria [34].

### 2.2. Study Design and Conduct

Data analysis was conducted in two phases. The study sample was* a priori* divided randomly into hypothesis development and validation cohorts using a 66% to 33% sample division. All endpoints were specified in advance of data gathering and analysis.

### 2.3. Anesthesia and Surgery

Anesthesia was managed per the attending anesthesiologist's preference. All patients underwent nonpulsatile hypothermic (30°–32°C) CPB. Perfusion was maintained at pump flow rates of 2 to 2.4 L·min^−1^·m^2^ throughout CPB to maintain a mean arterial pressure (MAP) at 50–80 mm Hg. Mean arterial pressure was adjusted during CPB using intravenous vasoconstrictors and dilators by bolus and/or infusion as required. The pump was primed with crystalloid, and arterial blood gases were measured every 15 to 30 minutes to maintain arterial carbon dioxide partial pressures of 35 to 40 mm Hg, unadjusted for temperature (*α*-stat), and oxygen partial pressures of 150 to 250 mm Hg. Use of agents with potential renal effects (e.g., intravenous dopamine, antifibrinolytic agents) was not regulated. The CPB circuit was primed with mannitol (50 gm of 20% solution), crystalloid solution (0.9% normal saline), and 500 mL albumin/hetastarch and packed red blood cells as required. Typically, acceptable hematocrit ranged from 22 to 24% during bypass, with red blood cell transfusion usually occurring when values below 20% were observed. Anterograde and retrograde blood cardioplegia delivered between 6 and 8°C was the myocardial protection strategy of choice.

### 2.4. Renal Function (Dependent) Variable Assessment

Preoperative and daily postoperative serum creatinine values were measured until hospital discharge as per institutional protocol. If more than one creatinine per day was measured, then the first measurement was used. Serum creatinine was measured using a dry slide enzymatic reflectance technique (Vitros 950, Johnson and Johnson, New Brunswick, NJ) with a normal range of 44–133 *μ*mol/L (0.5–1.5 mg/dL). Preoperative creatinine was obtained within one week prior to surgery and defined as the value recorded closest to but not on the day of surgery. The peak postoperative creatinine value was the highest of the daily in-hospital postoperative creatinine values. Preoperative to peak postoperative change in serum creatinine was the relative difference between preoperative and peak postoperative values (%ΔCr).

### 2.5. Blood Pressure (Independent) Variable Assessment

Following radial arterial line placement, intraoperative blood pressure measurements were captured every 30 seconds for all patients as part of their electronic medical record. In total nearly 1.5 million (1,495,289) blood pressure data points were evaluated. Rules were used to exclude implausible blood pressure values and those suggestive of artifact such as line occlusion or open-to-air. Systolic blood pressure values beyond 299 mm Hg or below 25 mm Hg were excluded from analysis. Blood pressure measurements during CPB were also excluded since nonpulsatile CPB perfusion pressure variation is dissimilar to blood pressure variation unrelated to CPB, and several studies including one from the study institution, [35] have failed to demonstrate any relationship between postcardiac surgery AKI and perfusion pressure where pump flow guidelines maintain calculated cardiac index.

For study purposes,* baseline blood pressure* was defined as the median of the first 5 intraoperative recorded values, occurring while the patient was awake and mildly sedated but prior to anesthesia induction. *Peak and nadir variables* were calculated from the most extreme intraoperative fluctuations in systolic blood pressure (SBP) compared to baseline blood pressure. *Excursion episodes* beyond various systolic threshold criteria (20% above, 20% below, and 20% above or below SBP baseline) were characterized by their occurrence frequency (total and number above and below), duration (cumulative total and mean episode duration), and “area under curve” (AUC), as reflected by an integral of degree and duration calculated in mm Hg·min units (total and AUC above and below), as previously described [36, 37].

### 2.6. Statistical Analysis

Patient characteristics were described using percentages for categorical variables and medians and interquartile ranges (IQR) for continuous variables. Assessment for associations between AKI (%ΔCr) and each derived measure of systolic blood pressure variability was then investigated using separate multivariable linear regression models, adjusted for aprotinin use, age, CHF, previous MI, baseline creatinine, bypass time, diabetes, weight, valve surgery, gender, and preoperative pulse pressure. No adjustment was made for multiple comparisons in these development analyses since all inquiries were considered exploratory. After thorough investigation in the development dataset, a single primary hypothesis was generated for testing in the validation dataset. Preoperative hematocrit and intra-aortic balloon pump utilization were tested separately as interactive variables with intraoperative systolic hypotension for an association with postoperative AKI. Secondary (e.g., cubic splines) analyses of the combined development and validation dataset were employed to further explore the validated significant associations. All analyses were performed using SAS statistical software (version 9.1.3, SAS institute Inc, Cary, NC). *P* < 0.05 was considered significant. The study was conceived and designed by Solomon Aronson; the data gathered and analyzed by Solomon Aronson, Mark Stafford Smith, and Barbara Phillips-Bute. The paper was written by Solomon Aronson and Mark Stafford Smith and edited by Solomon Aronson, Jeffrey Gaca, Manuel Fontes, Joseph P. Mathew, and Mark F. Newman. 

## 3. Results

Final development and validation samples included 4864 and 2383 patients, respectively, with complete blood pressure and renal data (Table 1). There were no differences in any of the descriptor variables between the development and validation cohorts. Inspection of systolic blood pressure distribution supported *a priori* criteria for exclusion, including 482 values above 299 (0.03%) and 11,839 below 25 mm Hg (0.79%), from a total 1,495,289 systolic blood pressure determinations. A priori it was decided that values above 300 and below 25 represent an artifact and were excluded. In contrast, values between 250 and 299 mm Hg were rare (0.1%) but fell within a normal distribution of systolic blood pressure values and were therefore not excluded.

Measures of systolic blood pressure variability (Table 2) were each evaluated separately for association with AKI (%ΔCr) in linear regression models, adjusted for covariates. Based on model *r* ^2^ value in the developmental sample, a marker of systemic hypotension relative to baseline was identified as the single best predictor of % delta creatinine (*P* < 0.006: model *r* ^2^ value = 0.065). This measure was calculated as systolic blood pressure drop from baseline to nadir, expressed as a percentage of baseline. Therefore, this single measure (% drop) was selected for validation in the validation sample.

**Figure 1:**
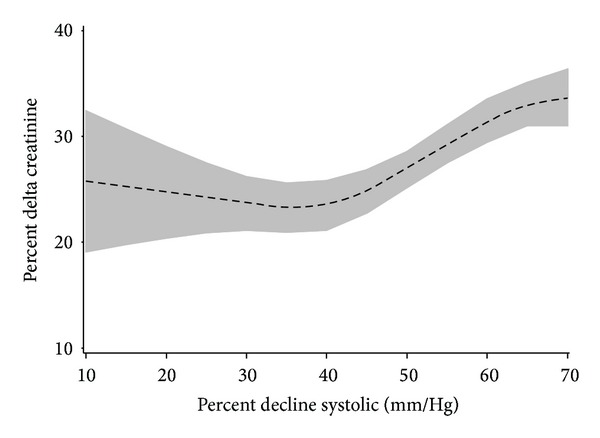
Association of % change in systolic blood pressure and percent decline in creatinine in the combined sample (*n* = 7504) Predicted values are derived from a multivariable linear regression model which incorporates restricted cubic splines. This allows the slope of the line to vary over the range of values on the *x*-axis to represent a nonlinear association. The shaded area represents 95% confidence intervals for the predicted values, which are represented with the dotted line.

In a multivariable linear regression analysis using the validation sample, an association between percent systolic blood pressure change below baseline and %ΔCr was validated (*P* < 0.0016) in the validation data set. Descriptive data, *P* values, and model *r*-square values for all of the tested measures are presented in Table 3. A nonsignificant interaction term was found to exist (*P* = 0.44) for the effect of SBP on renal function across the range of preoperative HCT values as well as utilization of an intraoperative aortic balloon pump (*P* = 0.25), Table 4. A secondary cubic splines analysis, using the combined dataset (development plus validation), of the relationship between percent systolic blood pressure change below baseline and %ΔCr (Figure 1) depicts the association, with 95% CI. The restricted cubic splines analysis (for descriptive purposes) illustrates overall nonlinearity of the association. Within the linear component of our model the slope of the linear regression indicates that for every percent decrease in SBP, there is a corresponding increase in % delta creatinine of 0.3%.

## 4. Discussion

 AKI as evidenced by small elevations in creatinine compared to baseline following cardiac surgery has been shown to predict increase in-hospital mortality and morbidity [11–16, 32, 33]. A strategy to identify and mitigate this modifiable risk factor for AKI may improve outcome. Among the most powerful predictors of AKI following cardiac surgery, most of them are not modifiable (i.e., age, gender, preexisting renal insufficiency, and other comorbidities), whereas our findings demonstrate a clinically significant association between otherwise clinically acceptable intraoperative systolic blood pressure reductions relative to baseline and postoperative elevations in creatinine. That there may be a threshold below which reductions of intraoperative systolic blood pressure predict subsequent renal dysfunction outcome is clinically provocative and raises questions on current clinical approaches to intraoperative blood pressure monitoring and treatment.

 The odds ratio for this association—although small—is very important for two reasons. First, intraoperative blood pressure is potentially modifiable. We believe that these data demonstrate the importance of intraoperative systolic blood pressure below a presenting baseline threshold that adds to the other recognized variables that predict AKI. Secondly, these data offer greater insight into the physiology of pulsatile blood pressure variability influence on target organ pressure-flow dynamics.

 Preoperative hypertension has long been recognized as an important predictor of perioperative outcome. In addition, hypertension specifically characterized by systolic, diastolic, and pulse pressure subtype has been appreciated as a more complex marker for specific underlying cardiovascular disease and postoperative adverse outcome [3, 4, 8, 25, 27, 28, 38–40]. These data provide evidence that in addition to preoperative BP risk markers, intraoperative systolic blood pressure targets may need to be more carefully considered as well based on presenting information about cardiovascular risk [41–50].

 Defining low intraoperative systolic blood pressure based on a percent change from baseline systolic blood pressure and not absolute values is intrinsically logical because of occult abnormal vascular biology in these patients which shifts their autoregulatory curve to the right [24, 26, 41, 42, 47, 50, 51]. Our data suggests that intraoperative systolic blood pressure decrease below baseline systolic blood pressure accounts for a significant association with increasing changes in creatinine even after accounting for other known risk factors and should give guidance to identify patients at risk for postoperative AKI. 

## 5. Limitations

 The patient population represented a “relatively homogeneous” population principally relating to history of preexisting hypertension with similar systolic and diastolic classifications as well as other common comorbidities seen in a surgical population electively scheduled for cardiac surgery. An interesting subanalysis would be to separate on-bypass from off-bypass procedures (as well as coronary bypass from valves procedures), but the small sample size of both off-bypass and valve procedures precludes this. This hypothesis sought to primarily explore the association of intraoperative systolic blood pressure variability with postoperative changes in creatinine. 

These data only represent intraoperative pulsatile blood pressure and exclude static mean arterial pressure (MAP) during cardiopulmonary bypass (CPB). While we acknowledge that blood pressure management during CPB is an important and interesting variable, we chose not to include the MAP index during CPB in this analysis because data regarding MAP during CPB and postoperative outcomes are unclear. Whereas Fischer et al. [52] reported that 4% of patients with normal preoperative renal function developed renal dysfunction after cardiac surgery with CPB particular when MAP was below 60 mm Hg during CPB, others could not validate these findings [35]. Gold et al. [53] compared two strategies of blood pressure management during CPB and concluded that high MAP may improve outcomes, but this finding could not be subsequently validated either. We maintain that blood pressure management during CPB is a fundamentally different, albeit important question that deserves separate discussion altogether. The question of confounding influence is also a separate question.

## Figures and Tables

**Table 1:** Patient characteristics and procedural and renal outcome.

	Development sample (4864)	Validation sample (*N* = 2383)
Age, median (IQR)	65 (56–73)	65 (57–73)
Male	68%	68%
Caucasian	86%	86%
History of hypertension	70%	70%
History of congestive heart failure	17%	17%
History of COPD	11%	11%
Previous myocardial infarction	42%	42%
Prior CABG surgery	18%	17%
Diabetes	33%	33%
Baseline systolic, mm Hg median (IQR)	141 (122–161)	141 (122–161)
Baseline diastolic, mm Hg median (IQR)	64 (56–72)	64 (55–72)
Preoperative creatinine, mg/dL median (IQR)	1.0 (0.9–1.2)	1.0 (0.9–1.2)
Preoperative hematocrit, % median (IQR)	40 (36–43)	40 (36–42)
Body mass index, median (IQR)	28 (25–32)	28 (25–31)
Intraoperative nitroprusside use	45%	45%
Parsonnet risk score, (54) median (IQR)	7 (3–12)	7 (3–13)
Surgery duration mins, median (IQR)	197 (132–286)	190 (131–288)
CPB duration mins, median (IQR)	106 (83–133)	109 (85–134)
Aortic cross-clamp time mins, median (IQR)	60 (45–80)	60 (46–81)
Aprotinin given	10%	7%
Intra aortic balloon counter pulsation	9%	8%
CABG only on pump (%)	86	86
CABG + valve	6	6
Off pump	8	8

Abbreviations: CABG: aortocoronary bypass surgery, COPD: chronic obstructive pulmonary disease, CPB: cardiopulmonary bypass, IQR: inter quartile range.

**Table 2:** Characterizations of blood pressure variability and association with percent delta creatinine*. *

BP descriptor	*P* value for BP measure	Model *r*-square	Hemodynamic variable median (25th–75th percentile) (units are indicated in the first column)
AUC for 95/135 (mm Hg·min)	0.33	0.058	930 (424–2707)
AUC < 95 (mm Hg·min)	0.36	0.058	447 (130–1584)
AUC > 135 (mm Hg·min)	0.72	0.058	207 (71–443)
AUC for 20% (mm Hg·min-above + below)	0.08	0.059	2268 (702–5698)
AUC for 20% below baseline	0.09	0.059	2117 (509–5542)
AUC for 20% above baseline	0.74	0.058	5.07 (0–74)
Number of total incursions	0.31	0.058	13 (9–17)
Number of incursions >135	0.25	0.058	4 (2–6)
Number of incursions <95	0.57	0.058	9 (5–13)
Cumulative minutes >135 or <95 mm Hg (min)	0.42	0.058	17.7 (3.1–6.8)
Minutes >135 or <95 mm Hg per incident (min)	0.29	0.061	4.8 (3.3–7.8)
Cumulative minutes >135 mm Hg (min)	0.39	0.058	14 (6–25)
Minutes >135 mm Hg per incursion (min)	0.84	0.061	3.7 (2.3–5.7)
Cumulative minutes <95 mm Hg (min)	0.55	0.058	49 (18–116)
Minutes <95 mm Hg per incursion (min)	0.27	0.058	4.9 (2.8–10.8)
Mean incursion nadir <95 mm Hg	0.01	0.060	13 (9–17)
Mean incursion peak >135 mm Hg	0.56	0.058	21 (12–31)
Percent change below baseline (to minimum)	0.006	0.065	53 (62–43)
Percent change above baseline (to maximum)	0.51	0.063	24 (9–48)

All models are adjusted for aprotinin use, age, chf, previous mi, baseline creatinine, bypass time, diabetes, weight, valve surgery, gender, and pulse pressure. *P* values for these covariates are not shown.

**Table 3:** Single hypothesis test in the validation dataset. Multivariable linear regression model showing association between % change in SBP and percent delta creatinine.

Predictor	Slope	*P* value
% SBP change below baseline	0.30	0.0016
Aprotinin given	23.58	<0.0001
Preoperative creatinine	−4.51	<0.0001
Total pump time	0.08	0.0001
Diabetes	9.37	0.0001
Weight (kg)	0.16	0.01

**Table 4:** Multivariable linear regression model showing association between % change in SBP and percent delta creatinine with interaction variable intraoperative balloon pump.

Predictor	*P* value
% drop in systolic BP	0.0261
Aprotinin given	<0.0001
Age	0.0112
Congestive heart failure	0.0132
Preoperative creatinine	<0.0001
Total CPB pump time	<0.0001
Weight (kg)	<0.0001
Preoperative pulse pressure	<0.0001
Preoperative HCT	0.0009
IABP use	0.0044
Interaction term SBP × IABP use	0.2525

## Abbreviations

AUC: Area under the curve
BP: Blood pressure
CABG: Coronary artery bypass grafting
DBP: Diastolic blood pressure
MI: Myocardial infarction
SBP: Systolic blood pressure.
